# Moralization and Mismoralization in Public Health

**DOI:** 10.1007/s11019-022-10103-1

**Published:** 2022-08-31

**Authors:** Steven R. Kraaijeveld, Euzebiusz Jamrozik

**Affiliations:** 1 Wageningen University & Research, Wageningen, The Netherlands; 2 Oxford-Johns Hopkins Global Infectious Disease Ethics Collaborative, Johns Hopkins University, Baltimore, United States; 3 Ethox and Wellcome Centre for Ethics and Humanities, University of Oxford, Oxford, United Kingdom

**Keywords:** Moralization, Mismoralization, Public health, Public health ethics, Moral psychology

## Abstract

Moralization is a social-psychological process through which morally neutral issues take on moral significance. Often linked to health and disease, moralization may sometimes lead to good outcomes; yet moralization is often detrimental to individuals and to society as a whole. It is therefore important to be able to identify when moralization is inappropriate. In this paper, we offer a systematic normative approach to the evaluation of moralization. We introduce and develop the concept of ‘mismoralization’, which is when moralization is metaethically unjustified. In order to identify mismoralization, we argue that one must engage in metaethical analysis of moralization processes while paying close attention to the relevant facts. We briefly discuss one historical example (tuberculosis) and two contemporary cases related to COVID-19 (infection and vaccination status) that we contend to have been mismoralized in public health. We propose a remedy of de-moralization that begins by identifying mismoralization and that proceeds by neutralizing inapt moral content. De-moralization calls for epistemic and moral humility. It should lead us to pull away from our tendency to moralize—as individuals and as social groups—whenever and wherever moralization is unjustified.

“At intervals Salinas suffered from a mild eructation of morality. The process never varied much. One burst was like another.“

—John Steinbeck, *East of Eden*.

## Introduction

Moral thinking pervades our lives (Joyce 2007). Moral norms that stipulate the moral acceptability or unacceptability of certain kinds of behavior are part of every human culture (Arutyunova et al. 2016). It has been theorized that human beings have an innate moral grammar, akin to a linguistic grammar, through which we analyze the moral structure of other people’s actions (Mikhail 2011). In short, we are deeply concerned with the morality of other people’s behavior, if not our own.

The range of actions considered to fall within the purview of morality can change over time. Moralization is the process through which preferences are converted into moral values, both for individuals as well as at the level of culture (Rozin 1999). Something that was previously considered morally neutral (i.e., neither morally good nor bad) can, through the process of moralization, take on moral properties and become endowed with moral significance (Rozin 1997). Norms can become moralized beyond the level of *ought* (i.e., a norm that one ought to Φ) to the level of *must* (i.e., a norm that one must Φ) (Morris and Liu 2015). When an issue is moralized, it is more likely to receive attention from governments and institutions, to encourage scientific research about the subject at hand, to provoke censure, to become internalized as values, to be transmitted as attitudes from parents to children, and to motivate the search for post hoc supporting reasons (Rozin 1999).

Moralization is often linked to health and disease. Many people have a preference for health and against disease or illness; yet, above and beyond such preferences, many diseases have been subject to moralization in particular contexts. Diseases like AIDS (Cochran 1999; Nzioka 2000), mental illnesses like depression (Scrutton 2015), and disabilities more generally (Brooks 2021) have been moralized historically and to the present day. A classic example of a habit that has become moralized over time is cigarette smoking. Smoking was originally a morally neutral habit—one preferred to smoke or one did not—and was even considered fashionable. As smoking increasingly came to be seen as damaging to people’s health, however, it also came to be considered immoral (Rozin and Singh 1999). Another health-related phenomenon that has been moralized in many high-income countries is obesity, which is often characterized as a moral failure (Ringel and Ditto 2019; Townend 2009). Moralization can also already occur at the conceptual level of health, for instance when the concept of health itself is presumed to override or take precedence over competing norms and values (Thomas 2019).

In some cases, moralization leads to the recruitment of emotions like disgust and anger. Moral vegetarians, for instance, find meat more disgusting than health vegetarians (Rozin et al. 1997). Eating meat is not universally considered to be a moral issue, but it can become moralized within societies as well as for individuals (Feinberg et al. 2019). Conversely, feelings of disgust and anger can sometimes feed into moralization processes (Case et al. 2012). According to an influential social intuitionist account of moral judgment, moral judgments arise from quick, automatic evaluations (i.e., intuitions) that are sensitive to experienced emotions like anger and disgust (Haidt 2001).^1^ People’s intuitive disgust response to biotechnological innovations are known as the ‘yuck factor’ or the ‘wisdom of repugnance’ (Niemelä 2011; George 2012). The basic idea is that our intuitive disgust-response to a particular act or idea—especially a novel one—tells us something about its moral import (Kass 1997).

Some research suggests that experimentally manipulating disgust affects consequent moral judgments, so that disgust has been conceptualized as a moralizing emotion (for an overview, see Pizarro et al. 2011).^2^ Disgust may have evolved at least in part to regulate decisions within the domain of morality (Tybur et al. 2013). Emotions like disgust and anger predict different aggressive responses to moral violations (Molho et al. 2017). Sensitivity to disgust appears to predict moral judgment independently of political ideology (Van Leeuwen et al. 2017). Disgust sensitivity has also been found to predict negative attitudes (e.g., xenophobia and stigmatization) toward out-group members (Faulkner et al. 2004; Navarette and Fessler 2006) as well as obesity stigma (Lieberman et al. 2012).

Moralization can be a positive force, for instance by holding bad actors accountable and increasing cooperation (Crockett 2017), by expressing group values and inhibiting deviant behavior (Sawaoka and Monin 2018); or, in the case of public health, by signaling that an issue is morally important (Verweij and Dawson 2007). Nevertheless, it often exacerbates social conflict and can lead people to dehumanize others (Fincher and Tetlock 2016). Moralization can have a pervasive negative impact on social cooperation; it can reduce society’s capability to attain important societal goals and can legitimize stigmatization (Täuber 2019). Moralizing health-related behavior in particular can undermine social cohesion and divide society through the stigmatization of those deviating from health-related moral norms (Täuber 2018). Health-related stigmatization can also lead to relational injustice (Haverkamp et al. 2018; Kraaijeveld 2021a).

Across societies, human beings are inclined to punish norm violations, both directly (e.g., through confrontation) and indirectly (e.g., through gossip and social exclusion) (Molho et al. 2020). Moralized norms, particularly in the form of collective sacred values, may lead individuals to engage in violent political action with costly social consequences (Ginges and Atran 2009). Moralization can lead to moral outrage, an emotion that motivates people to shame and punish wrongdoers, which has become especially widespread in online environments (Crockett 2017). Therefore, while moralization may sometimes have positive effects, it is neither intrinsically good nor necessarily associated with good outcomes (cf. Brady and Crockett 2019).

Moralization has primarily been studied descriptively by psychologists, in relation to questions about how, why, and when moralization occurs. Potential normative consequences of moralization tend to be circumscribed to specific instances and consequences of moralization (e.g., Skitka 2010). In this paper, we provide a systematic normative approach to moralization. We introduce and develop the concept of ‘mismoralization’ in order to diagnose morally inappropriate cases of moralization. Mismoralization, we argue, is when moralization is metaethically unjustified. We discuss three examples of what we consider mismoralized issues: the primarily historical case of tuberculosis, and two contemporary cases of COVID-19^3^ infection and vaccination status, which are giving rise to increasing moralization and stigmatization in public health. The mere fact that risks surrounding infection and (opting out of) vaccination have become highly vivid for Covid over the past years does not in itself justify moralization. One must carefully examine the pertinent facts and the (use of the) moral concepts at stake. Epistemic and moral humility is advised in the face of rapidly changing circumstances and states of scientific, medical, and technical knowledge. Finally, we outline potential ‘de-moralization’ strategies that may help neutralize the moral charge of mismoralized issues.

## Mismoralization

As a description of social-psychological processes, moralization in itself is neither good nor bad. In order to arrive at a judgement about the morality of moralization, a perspective on the moralization in question is needed. By means of analogy, misinformation is in one sense simply information—even bogus information falls under some description of information. When we call a particular text misinformation, however, we go beyond the text as a mere artefact that tells us something, and instead signal that the text is also false or inaccurate in an important way (Southwell et al. 2019).

Analogous to the information-misinformation example, whether moralization is appropriate or not requires a metaethical position on what makes it so that moralization is not ‘merely’ moralization. We conceive of metaethics broadly as the attempt to understand “the metaphysical, epistemological, semantic, and psychological presuppositions and commitments of moral thought, talk, and practice” (Sayre-McCord 2014). The metaethical position that is needed to diagnose mismoralization is directed at the moral thought, talk, and practice surrounding any moralized issue that contains moralized content and is associated with moral judgment (e.g., that X is bad, Y is wrong, Z is harmful, etc.). The literature on the nature of moral judgment is extensive, and we do not take a position on it here. For our purposes, moral judgments refer to “the rightness or wrongness of specific acts or policies,“ which can be driven by cognitive and affective processes (Waldmann et al. 2012, 274).

Generally stated, then, mismoralization is inappropriate (i.e., metaethically unjustified) moralization. Given that morality is the target of our analysis, it is important to say something about what makes an issue *moral*. A distinction is often drawn between descriptive and normative senses of the concept of ‘morality’, where the former refers to an accounting of “certain codes of conduct put forward by a society or a group,“ and the latter refers prescriptively to “a code of conduct that, given specified conditions, would be put forward by all rational people” (Gert and Gert 2020). While our analysis commences with a description of morality (i.e., the givenness of a moralized issue), we may arrive, through metaethical reflection, at the normative conclusion that an issue has been mismoralized. For our account, it does not matter whether the moralized issue was previously morally neutral, as it does for social-psychological theories. Morally speaking, what matters is whether or not a moralized issue ought to be morally neutral. When an issue that should not be morally charged is nevertheless inappropriately moralized, then this should be understood as a case of mismoralization.

**Fig. 1:**
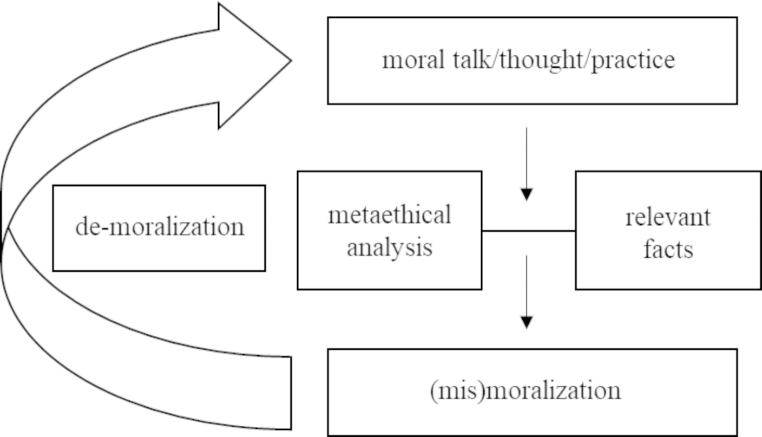
Diagnosing mismoralization.

Figure 1 provides a broad overview of how to diagnose mismoralization. At the top of the figure—at the heart of the matter—is moralized talk, thought, and practice, which constitutes the subject of analysis. Stepping outside of that subject to determine whether mismoralization is taking place requires metaethical reflection: Are the relevant moral concepts, moral arguments, and moral judgments valid? It also requires careful consideration of the relevant facts, which are both inherently and metaethically important. Theoretically, an analysis of mismoralization could be purely philosophical; for instance, by showing that a moral concept or argument on which people rely to arrive at a particular moral judgment is logically unsound. However, we are not as interested in challenging particular moral judgments as we are in challenging wider processes of moralization among groups of people. There are, after all, a number of ways in which moral practices can come unstuck from metaethical justification. Sometimes people are simply unclear or mistaken about normative issues (e.g., about what is right or wrong). Often, however, it is because (1) the set of relevant facts is incomplete or unknown, and/or (2) there is an appeal to irrelevant facts, and/or (3) the causal connections between pertinent facts are misunderstood or unknown. What is needed, then, is an account of the morally significant facts at hand in conjunction with sound metathetical reflection. Only then are we in a position to assess whether or not a moralized issue is, in fact, mismoralized. If we find that it is, then it is crucial to feed this conclusion back into the moralized issue that was the subject of analysis, in order to attempt to neutralize or 'de-moralize’ it.

Mismoralization, then, is identified when moralization is determined to be unjustified from a metaethical perspective and in light of the facts at hand. It should be noted that one can at least theoretically distinguish between the processes of moralization itself (i.e., when an issue takes on moral properties) and the consequences of this process (e.g., derogation or punishment of those deviating from the norm). Furthermore, some issues may be appropriately moralized upon reflection, yet moralization of the issue may still lead to morally unacceptable and/or instrumentally counterproductive outcomes (e.g., stigmatization, ostracism, social and political division, behavioral reactance, etc.). It may not always be practically possible to distinguish the metaethical judgment that a particular issue is a moral issue from the constellation of phenomena associated with moralization, including undesirable consequences (Rhee et al. 2019). When we speak of the mismoralization of an issue, we mean first and foremost the inappropriate moralization of that issue, although this will likely mean that the associated negative and/or harmful consequences are also morally problematic. Moreover, the degree to which moralization is appropriate may not necessarily track the magnitude of the detrimental effects that are associated with it. In cases where moralization is inappropriate, but where there are few or insignificant negative effects, mismoralization is arguably less problematic than in cases where the negative effects are rampant. This is especially true when mismoralization leads one party to harm others who may not even endorse the moralization in question.

## Mismoralization and Public Health

It is perhaps easier to understand complex social-moral issues retrospectively. From the vantagepoint of history, one gazes comparatively calmly at what happened in the past in order to make sense of it. On the contrary, when the dynamics of a given situation are in full force, analysis may be more difficult—especially for issues that are charged with moral and emotional significance. At the same time, when harms and injustices are at stake, one cannot delay one’s response until the fire of the moment has burned out. This is one reason why metaethical analysis can be invaluable. Like historical analysis, it means stepping away from the givenness of a particular situation, thus enabling the emergence of a larger perspective.

In what follows, we first discuss tuberculosis as a primarily historical example of public health mismoralization. Historical examples can help to remind us that present states of affairs are often not as unprecedented as commonly believed. We then proceed to discuss the moralization of Covid infection and vaccination status, which, we argue, are currently mismoralized.

### The Antituberculosis Movement and Sanitary Science

Before 1880, tuberculosis (TB) or ‘consumption’ was seen by doctors and laymen alike as a hereditary disorder.^4^ However, as the germ theory of disease gained wider acceptance in the 1880s, it came to be understood as a chronic communicable disease, and hygienic infractions “once regarded as merely disgusting or ill-bred, such as indiscriminate spitting or coughing, now became defined as serious threats to the public health” (Tomes 1997, 272). The new understanding of TB as an infectious disease resulted in increasingly pervasive and aggressive public health campaigns to prevent its transmission—the so-called sanitary science and ‘antituberculosis’ movements. As TB-related behavior was no longer restricted to the individual domain, it increasingly took on social-moral dimensions as part of a widespread process of ‘moralizing the microbe’ (Tomes 1997).

Consumption has a longer history of being moralized—not in terms of public health, but for the individual sufferer. In *Illness as Metaphor*, Susan Sontag (1990) writes of the mythological and metaphorical representations of consumption in the works of writers and artists. The ‘passion’ to which consumption was thought to give rise is brilliantly described in works by authors like Fyodor Dostoevsky and Ivan Turgenev. One of the best descriptions of the moral dimension of suffering from (consumptive) disease, however, comes from Thomas Mann in *Doctor Faustus*: “Disease […] creates a certain critical opposition to the world, to mediocre life, disposes a man to be obstinate and ironical toward civil order, so that he seeks refuge in free thought, in books, in study” (Mann 1997, 248). Endowing a disease like TB with moral properties for the sufferer is clearly a very different kind of moralization from that which began after the 1880s, when TB was no longer seen as a disease of the *infected* but as a disease of *the contagious*. It no longer only affected the *self*; it could now also afflict *others*.

Clearly, the fact that a disease is infectious raises legitimate moral considerations and duties, like the obligation to make sure that one takes reasonable precautions not to infect other people (Verweij 2005). Yet the infectious nature of TB, once understood, gave rise to an anxious fervor among scientists and public health officials in the late nineteenth and early twentieth centuries that culminated in what has been described as a pseudo-religious approach: TB workers “invoked religious language and symbols in their hygienic exhortations,“ and used recurrent terms such as “salvation, gospel, and crusade,“ which permeated their work with “a sense of spiritual mission” (Tomes 1997, 278). The originally Christian emblem of the double-barred cross, for example, became the international symbol of the fight against TB.^5^ To counteract the ever-present danger of contagion, health education and educators employed a heavy-handed moral approached and pushed a “sort of moral regeneration,“ as one anti-TB worker described it, which aligned TB control with the virtue of temperance and with mental hygiene (Tomes 1997, 282). The “TB crusade” at the time disproportionately targeted women, who were held to be responsible for the welfare of the home, as well as poor people. It appealed to common stereotypes of the ‘other’ as dirty and dangerous, leading to harsh condemnation of those who contracted the disease (Tomes 1997). Neither targeting women for supposed failure in housekeeping duties nor blaming the poor for contracting TB—when the poor were forced to live in conditions that made contracting the disease much more likely—were morally justified from a metaethical perspective. While moral condemnation may have been appropriate in some cases, for instance if a person purposefully infected others,^6^ the moralization of infection *as such* was unwarranted, especially when such condemnation was tied to poverty or gender roles, which are not morally relevant categories in themselves.^7^

Tuberculosis stigma persists to this day, especially in TB-endemic, low-income countries where the disease is prevalent and where it is associated with shame, isolation, and fear (Juniarti and Evans 2009). The widespread stigmatization of TB is often a result of the ways in which the disease is moralized; for instance, as a ‘dirty disease’ (still) linked to immoral practices and bad behavior (Long et al. 2001) or to divine punishment for a moral failing (Baral et al. 2007). The associated stigma is a barrier to TB control and to TB prevention, care, and treatment (Courtwright and Turner 2010; Datiko et al. 2020). Moralization of TB is not justified from a metaethical perspective, because (1) it is not caused by immoral behavior or practices, (2) infection risk is determined by more than individual behavior, which means that seeing individual behavior as the sole determinant of risk is unjust, (3) the risk to others appears to be significantly smaller than commonly believed, because most people with TB are asymptomatic and not contagious. Moreover, given that the harms of moralization (e.g., through stigmatization, shaming, and fear) for both individuals and for public health are potentially very high, there are at any rate pressing instrumental reasons to avoid moralizing TB infection (WHO 2017).

Once TB was understood to be communicable disease and became moralized, the stage was set for the moralization of other infectious diseases. In the following section, we address the moralization of COVID infection.

### Covid Infection Status

Moralization has been widespread during the Covid pandemic (Graso et al. 2021). Moralizing language related to Covid has been common in news media (Malik et al. 2021). Mitigating practices at an individual level, such as hand-washing and social distancing, have been widely communicated as moral imperatives, leading to interactional tension between those who strictly adhere to mitigating practices and those who do not—e.g., between ‘distancers’ and ‘non-distancers’ (Prosser et al. 2020). Physical distancing, for instance, was found to predict moral condemnation across a number of different countries (Bor et al. 2020).

Like TB infection, Covid infection is part of the larger phenomenon of ‘moralizing the microbe’ that potentially gives rise to stigmatization. Since the early days of the pandemic, people have been blamed and shamed for getting Covid, which has led to embarrassment about, for instance, having to share a positive test result. We witnessed the manifestation of moralization and social stigma surrounding Covid infection, which only seems to be intensifying as the pandemic persists (Bagcchi 2020; Grover et al. 2020; Abdelhafiz and Alorabi 2020).

It was only in December of 2021 that an opinion article emerged in the mainstream news media that explicitly urged people to stop blaming others for getting Covid (Olen 2021). The article took issue with a narrow focus on individual responsibility for not getting infected and the persistent blaming and shaming of people who did and do get Covid. We will not address the issue of government as opposed to individual responsibility here. Rather, we want to step back and ask the following question: Is moral blame directed at other people for getting infected with Covid justified?

At the beginning of the pandemic, the prospect of avoiding infection might have seemed like a realistic goal. We did not yet know how far SARS-CoV-2 would spread. We also did not know at that time whether it would become an endemic virus that might face us for the rest of our lives—although it should be noted that past pandemics typically became globally endemic, and that vaccines enabling elimination of respiratory viruses have proved difficult to develop in the past (Heriot and Jamrozik 2021). At the present time, however, consensus is forming that SARS-CoV-2 cannot be eradicated and, like other seasonal coronaviruses, is becoming an endemic virus, so that everyone stands to get infected at least once during their lifetime (Philips 2021; Veldhoen and Simas 2021).

With this in mind, let us reflect on whether moral condemnation and blame are appropriate for getting infected with Covid. In order for moralization to be justified, we have to determine when people are morally responsible for their actions and the events that they bring about (Talbert 2019). There are two necessary and jointly sufficient conditions—an epistemic condition and a control condition—under which a person may be considered morally responsible (Rudy-Hiller 2016). The epistemic condition, on the surface of it, largely appears to hold. By now, the public generally knows under which conditions the risks of getting infected with Covid are highest—although it is by no means certain that one can always know how to prevent infection. With ongoing scientific debates among experts about, e.g., surface transmission, aerosol transmission, the effectiveness of different kinds of masks, and so on, it is probably unreasonable to expect laypeople to be guided in their actions by anything like a full-fledged understanding of Covid transmission dynamics. Nevertheless, let us grant the epistemic condition for the sake of argument, and assume that it holds.

What about the control condition? Are people generally in a position where they can exercise control over whether or not they get infected? Given the ubiquity of the virus and the prospect of endemicity, this hardly appears to be the case. One could attempt to delay infection by taking far-reaching precautions (cf. Verweij 2005). But, even if one were to virtually seclude oneself in one’s house for the rest of one’s life, there are still bound to be opportunities for infection to occur. One needs food, after all—there must be some contact with the outside world, with one’s family and friends. It is not realistic to expect that one can avoid infection for the rest of one’s life; nor that one always has control over whether or not one gets infected in the long run. Of course, there are behaviors that can significantly increase one’s risk of infection. This seems to be what is taken as a morally significant component of much moralization surrounding infection. However, the level of ‘riskiness’ of behavior as far as one’s own self is concerned is ultimately more of a question about *surface* rather than *deep* control. On the face of it and for the time being, one can exert some control over infection status by abstaining from behaviors that carry relatively high risks. Nevertheless, unless one is willing to dedicate one’s life to the avoidance of Covid infection—and even then—there is no deeper sense in which one can realistically have control over getting infected with endemic respiratory viruses.

In light of this, we have to ask the following question. Why would people, even if they engage in what might be considered higher risk behaviors (e.g., going to bars, concerts, or gyms) deserve to be morally blamed or shamed, when everyone stands to be infected in the long run, including more cautious and risk-averse people? Furthermore, importantly, we are speaking here of *getting* infected—not of *spreading* infection.^8^ Therefore, the relevant target of comparison for getting infected is engaging in other activities that can potentially cause harm to oneself—many of which are morally acceptable in myriad ways and across many areas of life without being associated with blame or shame (e.g., playing extreme sports, driving a car, etc.). What is the moral principle, then—the metaethical justification—that would meaningfully differentiate the risk of getting infected with Covid through regular activities from many similar cases, like getting infected with seasonal influenza? No such principle appears to hold.

From a metaethical perspective, then, and while taking into consideration the epidemiological facts, blaming other people for getting infected is unwarranted. Nor, it should be added, is blaming oneself, which is also becoming a serious psychological problem—for instance, through feelings of guilt when people find that they are unable to personally meet the moralized notion of ‘staying healthy’ (Lane 2022). Getting infected with Covid over one’s lifetime is highly likely; one can take reasonable precautions (e.g., by getting vaccinated), but even getting vaccinated will not prevent one from getting infected with Covid (Singanayagam et al. 2021). After all, if a group of fully vaccinated and meticulously tested workers in a Belgian research station in remote Antarctica can get Covid, then anyone can (Kekatos 2022). It is time to let go of the mismoralized charge and the stigma surrounding Covid infection.

This brings us to the final case, namely the ongoing moralization of Covid vaccination status, discussed in the next section.

### Covid Vaccination Status

Arguably the most deeply moralized issue during the pandemic has been vaccination status. Whether or not someone has gotten vaccinated against Covid has taken on acute moral significance within many scoieties. The moralizing phrase ‘pandemic of the unvaccinated’ was coined early on in the pandemic and quickly caught on. The idea that the pandemic has become more or less exclusively the domain of unvaccinated people has been persistent.

Yet, while in many countries it was true that, at the beginning of vaccine rollouts, more unvaccinated than vaccinated people were hospitalized, this is no longer true in most highly vaccinated populations. In England, for example, the latest Covid surveillance report shows that the majority of hospitalized patients are fully vaccinated (Public Health England 2021). This makes sense, given that in highly vaccinated populations, the absolute number of hospitalizations among the relatively much bigger group of fully vaccinated people is likely to be larger than the absolute number of hospitalizations among the relatively much smaller group of unvaccinated people, and given that vaccines provide imperfect protection against hospitalization (Tenforde et al. 2021).

Not only is it factually inaccurate to speak of a pandemic of the unvaccinated in highly vaccinated populations, but it is also likely to be harmful. By signaling to vaccinated people that they are prevented from experiencing illness, vaccinated people may come to behave in ways that paradoxically could increase their risk of infection. Furthermore, the current Covid vaccines neither provide sterilizing immunity (Vashishtha and Kumar 2022) nor prevent transmission (Federman 2022; Wilder-Smith 2021), so that vaccinated people can still be infected and spread infection to others. Recent studies, for instance, have found that fully vaccinated individuals with breakthrough infections have peak viral load similar to unvaccinated individuals and can efficiently transmit infection (Singanayagam et al. 2021; Acharya et al. 2021). Breakthrough cases among groups of fully vaccinated people are becoming more common to the point where they are now widely reported in the news (Christensen et al. 2021; Quiroz-Gutierrez 2022). In sum, vaccinated people continue to have as relevant a role in SARS-CoV-2 epidemiology and infection transmission as unvaccinated people, which has led to a call on high-level officials and scientists to end the inappropriate stigmatization of unvaccinated people (Kampf 2021).

Getting vaccinated against Covid seems to be primarily a self-protective choice at this point (cf. Kraaijeveld 2020a), yet other-directed considerations appear to be driving the moralization of vaccination status. Individuals arguably have a moral obligation to take precautions against infecting others (Verweij 2005), which may involve getting vaccinated. Yet it is by no means clear that (1) by not getting vaccinated one will thereby harm someone else, or (2) that by getting vaccinated one thereby avoids harming others. While getting vaccinated may decrease one’s chances of infecting others to some degree, and while the increased chance of hospitalization for unvaccinated people may potentially indirectly affect others through the utilization of scarce healthcare services, neither appears to warrant the strongly moralized verdict that a person harms others by not getting vaccinated. There are many small risks of harm to others that people permissibly take on a daily basis (Hansson 2003).^9^ The fact that risks pertaining to vaccination and infection have become so vivid for Covid, does not inherently justify moralization.

Not causing harm appears to be the primary moral principle at stake when it comes to the morality of getting vaccinated. Given that the current Covid vaccines neither prevent infection nor transmission, this makes harm-based moralization of vaccination status questionable. It is unclear at best that, under similar conditions, someone who has not gotten vaccinated is more likely to infect and/or harm someone compared to someone who has gotten vaccinated. This is especially true for unvaccinated people who have already been infected with Covid, because previous infection offers at least as much protection against hospitalization and mortality as do the vaccines (Kojima and Klausner 2022; Kim et al. 2021). For unvaccinated people who have recovered from previous Covid infection, the idea that they are driving the pandemic never did make sense.

The upshot, then, is that the morally relevant principle of not harming others is complicated by current conditions related to the available vaccines and to Covid transmission dynamics. The claim that unvaccinated people are directly threatening the lives of others, which feeds into vaccine status moralization, is very weak. The idea might be driven in part by people’s fears of infection. Self-interest was found to predict moral condemnation of other people’s behavior during the Covid pandemic across a number of different areas, including vaccination (Bor et al. 2021). The highest score on a measure of personal concern was associated with a 29% increase in moralization of vaccination compared to the lowest score, and with a 41% increase in condemnation of non-vaccination (Bor et al. 2021).

Disease-avoidance mechanisms can influence social cognition. People are more inclined to perceive out-group than in-group members as infectious or contaminating and to respond to them with disgust; the more vulnerable people feel to disease, for instance, the more disease-related stereotypes against immigrants they hold (Faulkner et al. 2004; Kelly et al. 2010). Although we are not aware of a study to this effect, it can be assumed that many people have come to feel vulnerable to disease during the Covid pandemic. Most people in high-income countries are fully vaccinated at this point, which makes for a large in-group of vaccinated people and a small but substantial unvaccinated out-group. From the perspective of vaccinated people, what might be driving some of the strongest moralization is precisely this mechanism of seeing the out-group (i.e., unvaccinated people) as more infectious and contaminating than they really are. A distance-keeping, diligently quarantining, unvaccinated person, particularly if she has already undergone a SARS-CoV-2 infection, seems to pose little threat to others given what we currently know. Yet such a person will presumably be included in any blanket moral judgment about the unvaccinated as harmful to society.

Unvaccinated people have been widely blamed for (1) prolonging the pandemic, and (2) the emergence of SARS-CoV-2 variants. The first notion does not hold up when examined in light of the latest data, which finds no correlation between percentage of the population vaccinated and Covid infections (Subramanian and Kumar 2021). More data is always needed, but we have at least a *prima facie* reason not to assume that, if only more people (i.e., a greater percentage of populations) were to get vaccinated, then the pandemic would be over. The end of a pandemic is, in any case, also a political decision, without a clear-cut or universal definition; it depends on many factors, not all epidemiological (Robertson and Doshi 2021). Blaming unvaccinated individuals and condemning them for prolonging a pandemic whose end is not clearly defined is misguided.

As to the second notion, the idea that unvaccinated people are responsible for new variants is tenuous at best (Wang, Chen, and Wei 2021). Given that there are animal reservoirs for SARS-CoV-2 (Valencak et al. 2021), that it is unlikely that all human beings around the world will concurrently get vaccinated against Covid (especially with waning vaccine immunity), and given that current Covid vaccines do not provide sterilizing immunity (Vashishtha and Kumar 2022), directly blaming unvaccinated people for the emergence of variants appears to be mistaken. The best available evidence does not support the idea that the unvaccinated are substantially causing or spreading novel variants. Perhaps it is wise, as it probably always is, not only to show epistemic humility in a novel situation marked by rapidly changing circumstances and states of scientific knowledge, but also to exercise moral humility by avoiding moral judgment and condemnation at least before one has a better understanding of the state of affairs. This goes for governments and public health officials as well as for individuals.

As for the recruitment of negative emotions and stigmatization, consider the following two prominent examples. First, French President Emmanuel Macron recently declared his nation’s roughly 5 million unvaccinated people to be ‘non-citizens’, claiming that he wanted to anger the unvaccinated by squeezing them out of public spaces (Onishi 2022). This open and targeted discrimination of a large group of citizens by an elected leader is truly unprecedented and, in our view, morally reprehensible. Second, Canadian Prime Minister Justin Trudeau recently questioned whether the rest of Canada should ‘tolerate’ the unvaccinated, furthermore suggesting on television that people who refuse to get vaccinated are often racist and misogynistic extremists (Naylor 2021). While Trudeau perhaps did not mean to imply that all unvaccinated people are racist and misogynistic, publicly associating a heterogenous group of unvaccinated people with some of society’s most unwelcome beliefs and behaviors is morally problematic. It directs people’s widespread hatred and disgust for racism and misogyny—and for those guilty of it—toward unvaccinated people. Even if it is true that some unvaccinated people are also racist and misogynistic, it is clearly unjustified to extend these pejorative terms to the overall group. Some vaccinated people are surely also racist and misogynistic—especially when most adults in Western societies are by now fully vaccinated (just consider the odds). Yet, associating people who got vaccinated with being racist and misogynistic is clearly unwarranted. Why would the reverse judgment be any more acceptable?

Anger and disgust toward the unvaccinated may take on extra force when these people are not merely unvaccinated, but ‘also’ bad in other ways. This slippery slope must be resisted. Leaders would do well to avoid engaging in it, no matter how politically advantageous it might be. The principle of tolerance lies at the heart of liberal-democratic societies. We live together even with people with whom we disagree—even with people whose behavior may run counter to our own. For the reasons outlined above, many of the charges that have popularly been leveled at the unvaccinated do not appear to hold. To the extent that the present Covid vaccines reduce one’s chances of hospitalization and death, one may lament that some people choose not to avail themselves of the vaccines. But we must move beyond discrimination based on medical decisions; beyond simplistic portraits of angels and demons, the vaccinated and the unvaccinated. Especially if we accept that Covid will be with us for a long time, we should be very careful how we moralize if we wish to maintain the delicate fabric of civil society, and if we want to be able to live together in the long run.

Finally, moralization of vaccine status may lead people to accept coercive measures like vaccine passports without fully realizing their limited efficacy or costs. A recent study of the number needed to exclude (NNE) for vaccine passports suggests that at least 1,000 unvaccinated people likely need to be excluded to prevent one SARS-CoV-2 transmission event in most settings (Prosser, Helfer, and Streiner 2021). Although we do not have data about how effective people generally believe vaccine passports to be (which, it should be noted, is their sole public health rationale), we suggest that the most ardent proponents of vaccine passports are likely to overestimate their effectiveness when it comes to preventing infection and disease.^10^ This might be partly because studies like the proceeding one have come out only very recently and will not be known to many; but it may also be because preventing SARS-CoV-2 transmission is the only publicly acceptable reason for excluding vaccinated people from society. Those who may privately wish to exclude the unvaccinated for other reasons—out of blame, resentment, hatred, and so on—are bound to nevertheless publicly espouse infection and disease prevention as a rationale. Unless, of course, like President Macron, they feel comfortable enough to simply state that they want to exclude unvaccinated people for reasons not directly related to public health.

## De-moralization

When moralization of a public health issue is judged to be wrong, steps must be taken to address it. It is worth re-emphasizing the potential significance of moralization for individuals and societies. When issues become moralized, this can lead to (1) greater attention from institutions and governments, (2) increased scientific funding and research, (3) heightened societal acceptance of censure and/or punishment, (4) internalization of relevant moral attitudes, (5) enhanced parent-to-child transmission of relevant moral attitudes, (6) increased motivation to search for post hoc supporting reasons, and, in some cases, (7) recruitment and increased employment of emotions like disgust and anger (see Rozin (1999) for an overview). Given the inappropriateness of mismoralization, what can be done to redress it? Here we propose de-moralization as a broad normative response to mismoralization.

Within the psychological literature on moralization, several opposing processes have been suggested (Rhee et al. 2019; Rozin 1997; Tenbrunsel and Messick 2004), all of which involve first studying the factors that lead people and societies to no longer consider an action or issue to be morally significant. This work has been linked to so-called ethical blind spots, which occur when people act against their own moral compass through unintentional unethical behavior (Sezer et al. 2015). Acting on incorrect priors about causality and harm (of which people may be unaware), for instance, may lead people to engage in behavior that they would otherwise consider unethical (e.g., public shaming of others). Research shows that people often maintain an ‘illusion of objectivity’—an incorrect view of themselves as being more objective in their judgments than other people (Chugh et al. 2005; Epley et al. 2006). Biases in moral judgment may go unnoticed; the literature on the implicit biases that we all share by virtue of our human psychology is vast (for an overview, see Brownstein 2019). People often fail to recognize the harm that implicit favoritism of social in-group members causes to members of social out-groups (Sezer et al. 2015). Not recognizing in oneself that one is engaging in mismoralization, for example by unjustifiably condemning outgroup members, may constitute an ethical blind spot.

According to a recent push-pull model of moralization, a given moral attitude represents an equilibrium between two opposing processes—those that ‘push’ toward greater moralization and those that ‘pull’ away from moralization (Feinberg et al. 2019). The outcome of the dynamics between these conflicting processes is that people tend to reach an equilibrium about how much (if any) they moralize a given action or issue. The moralization process often begins with “a particularly evocative stimulus that arouses strong emotions and cognitions that in concert signal possible moral relevance; the more strongly people experience these emotions and cognitions, the more likely they are to perceive the stimuli as morally relevant” (Skitka et al. 2021, 585). A particularly significant factor in public health ethics and in light of our previous discussion is perception of harm, which often pushes people toward greater moralization. Greater perception of harm has been found to significantly increase moralization and moral conviction over time (Wisneski et al. 2020). It is therefore especially important for discussions in public health to be based on realistic estimates concerning risks of harm to third parties. Exaggerated claims about risks of harm to others, especially by trusted public sources, are likely to intensify mismoralization.

Aside from moral emotions and perceptions of harm, connecting an issue with one’s existing fundamental moral principles or ‘moral piggybacking’ may push toward greater moralization (Feinberg et al. 2019). Social learning processes have been shown to amplify moralization (i.e., by increasing moral outrage) in online settings (Brady et al. 2021), so that combating moralization may require addressing such learning processes in ways that lead people to pull away from—rather than push them into—moral outrage. Additional research on what leads people to withdraw from moralization will also help increase our understanding of how to de-moralize mismoralized issues, and how to disrupt inappropriate moral equilibria that may have become crystalized. In fact, overcoming surplus moral constraints is sometimes required for human emancipation, and is arguably an important dimension of moral progress (Buchanan and Powell 2017).

To extend the earlier misinformation analogy, perhaps people can be inoculated against mismoralization in ways similar to those that have been proposed against misinformation. For instance, one might preemptively warn people against misleading moralization tactics (cf. Van der Linden et al. 2017) to avoid an issue from becoming unduly moralized in the first place. Attention to the use of language is one important way in which preemptive action could be taken against mismoralization, for example with regard to framing effects. Persuasive emotional frames, particularly those that recruit emotions like anger and disgust, are known to increase moralization (Clifford 2018). Issues are significantly more likely to become moralized and politicized when they are framed in ways that elicit anger and disgust; the effect of a single exposure to persuasive emotional frames was found to last at least two weeks (Clifford 2018). Emotional framing in the media, both traditional and social, is likely to exacerbate moralization. Moral-emotional language, for instance, was found to shape the diffusion of moralized content within social networks (Brady et al. 2017). The language in which issues are framed in media and other public communication channels should steer clear of emotional frames, especially those eliciting anger and disgust, in order to allow people to refrain from mismoralization.

Addressing normalization processes may also be important as potential de-moralization strategies.^11^ People’s beliefs about normality play an important role in social and moral cognition (Cialdini et al. 1990). Research suggests that people’s beliefs about what is ‘normal’ incorporates representations of statistical norms (e.g., the average) and representations of prescriptive norms (e.g., the ideal) (Bear and Knobe 2017). Casting the normality of moralization into doubt for a given issue, for instance by showing people that the way in which it has been morally framed is not commonly accepted (or not as commonly accepted as believed), can be an important way to recalibrate beliefs about the norm in question. There also appear to be robust cross-cultural differences in how certain kinds of social transgressions are moralized (e.g., Berniūnas et al. 2021), which opens up a way to potentially counter mismoralization within a particular society by showing how the subject is differently experienced elsewhere.

Labels that lump together heterogenous groups of individuals and reduce them to a single category are generally considered to be morally questionable, if not wrong; especially when such labeling leads to stereotyping and discrimination (Angermeyer and Matschinger 2005; Link and Phelan 2012). It is therefore surprising that terms like anti-vaxxer, with clearly pejorative (moral) connotations, has become so widely accepted throughout the Covid pandemic. While the general public sentiment is certainly against anti-vaxxers and in favor of ‘anti-anti-vaxxers’ (Bernstein 2021), this should not come at the cost of over- or mis-applying the term. Especially when leveled at people who, upon reflection, deserve no such epithet, labeling someone an anti-vaxxer not only stands to become meaningless (i.e., when the term has become so broad that it includes anyone who voices any kind of criticism related to vaccination policies), but it can also have detrimental consequences for society and even for the practice of science itself. When we dismiss as anti-vaxxers scientists who widely endorse vaccination but who have voiced specific concerns (e.g., about certain risks associated with mRNA vaccines), or academics who have criticized specific policies on ethical grounds (e.g., mandatory vaccination policies), this serves to stifle the potential for healthy and necessary debates. As a result, scientists may become more hesitant to publish certain kinds of research (e.g., about mRNA vaccine adverse events) for fear of being ostracized. Yet it should go without saying that it is fundamental not only to the enterprise of science, but ultimately also to the health and well-being of all people—including those who would dismiss or berate anyone voicing vaccination-related concerns—that such research should be freely conducted and disseminated after having been assessed in proper scientific ways.^12^ Widespread moralization that targets individuals through labeling, with the recruitment of emotions like disgust and anger, is ultimately deeply unhelpful; especially when directed at individuals who voice concerns in good faith or with at least *prima facie* legitimacy.

As for the potential consequences of such labeling, a number of psychological studies show that forceful messages and stigmatizing images in public health communication can lead to psychological reactance (a motivational state of resistance) and thus bring about the opposite effect of the intended behavioral change (e.g., Scheppert, Blechert, and Stok 2020). People subjected to stigmatization tend to show psychological reactance and the drive to reestablish autonomy after it has been constrained, often in unexpected and potentially counterproductive ways (Brehm 1966). People living with HIV, for example, showed strong psychological reactance to all forms of HIV-related stigma, which was furthermore positively associated with symptoms of anxiety and depression (Brown et al. 2016). Dismissing people who are hesitant or who have legitimate concerns about Covid vaccination by stigmatizing them in public discourse is bound to achieve more harm than good—as with any kind of pejorative health-based labeling. More generally, abstaining from the use of stigmatizing labels in public debates and on social and traditional media may be a vital part of de-moralizing public health issues.

Governments and public health institutions should therefore be mindful of the language and the moral-emotional frames that they use when communicating about potentially moralized issues. We ought to be reminded that moralization and its forces of “moral indignation and moral fervor,“ although common in public health, do not provide good guides for policy-making (Schmidt 2015, 25). Particularly during pandemics, when social coherence is a crucial collective good, and when faring well throughout and beyond the pandemic depends on the goodwill of a great many individuals, it is important as a matter of public health policy not to moralize—and to de-moralize whenever and wherever needed. There are in any case compelling reasons to resist moralization, particularly in the form of responsibility-indicating interventions, when it comes to health promotion (Brown 2018). But it is an especially pressing moral issue when, as we have argued, such moralization is shown to be morally inappropriate.

While governments can relatively straightforwardly adopt non-moralizing approaches to public health communication, one might think that it is difficult if not impossible for individuals to disengage from mismoralization. Convinced (mistakenly so) that a certain action is morally wrong, individuals gripped by anger and disgust might not be able to control their emotional—or behavioral—responses. This is indeed a challenging issue, but a useful parallel can be drawn to work on retributive intuitions (i.e., our rapid, automatic evaluations that someone or something must be punished) in cases of harm caused by artificial agents without clear candidates for blame. Drawing on literature about implicit biases, Kraaijeveld (2020b) has argued that, when people experience retributive intuitions that turn out to be metaethically unjustified, and when direct control over such intuitions is not feasible, moral responsibility can (and should) still be taken in the form of indirect control. For example, one might take indirect control of one’s intuitions through implementation intentions, which are a type of explicit instruction to oneself that, in a certain scenario X, one will not Φ in response to one’s experienced emotions or intuitions (see Webb et al. 2012). To make this more concrete, in the case of vaccination status mismoralization, for instance, this might involve telling oneself that the next time one reads about an unvaccinated person on social media, one will abstain from moral condemnation and resist the urge to respond in the language of anger. In this way, even if direct control over how one reacts emotionally to an event is difficult, indirect control might still be taken. Ideally, perhaps, top-down forces (e.g., at the level of government and public health institutions) help to ensure that inappropriate public health moralization does not flare up at the level of individuals. This does not mean, however, that individuals cannot take responsibility for themselves. We all should. Epistemic and moral humility are eminent virtues. Once we recognize that an issue has been mismoralized, we may, to paraphrase Franz Kafka, have no choice but to let our hate- and disgust-filled head rest on our chest.

While we have focused in this paper on mismoralization of public health, mismoralization is potentially relevant to many other areas. Technological developments in science and medicine can and have in many cases become moralized (e.g., Shah and Boelens 2021; Newman 2012; Ricart and Rico 2019; Mihailov et al. 2021). Human-robot interaction is another area in which moralization may occur (see, e.g., Nyholm 2020; Mayor 2018) and where such moralization may not always be justified. Some have argued that technological change is impacting the role of disgust as a moral emotion in the Anthropocene (Thiele 2019), which may have important consequences for moralization processes. Mismoralization appears to lie in wait especially for novel technologies with potentially significant social-moral ramifications, for instance in the case of socially disruptive technologies (Hopster 2021).^13^ When moralization of an issue is particularly powerful and pervasive in society, identifying and opposing it as mismoralization may require considerable effort and not a little courage.

## Conclusions

Human beings are moral beings and as such are prone to moralization. We sometimes endow previously morally neutral issues with moral properties, which can be apt, but which is often detrimental to public health and to society. We introduced and developed the concept of mismoralization, which is when moralization is judged to be morally wrong. Metaethical reflection is necessary in order to identity cases of mismoralization. We discussed three cases that we believe exemplify mismoralization: the historical example of tuberculosis, Covid infection, and Covid vaccination status. Given that mismoralization is morally inappropriate, it is imperative that it should be either avoided or remedied, especially to the degree that it negatively affects social cohesion and leads to the stigmatization of individuals. We proposed de-moralization as a corrective process, which involves actively working against the forces that lead to mismoralization, like avoiding or opposing emotional frames eliciting disgust and anger. Pulling away from what makes us moralize is a psychological process. But it is also a moral process with major social implications; there is a larger sense in which we ought to eschew mismoralization, as individuals and as societies.

## Footnotes

1 But see Kasachkoff and Saltzstein (2008) for a critique of the social intuitionist model.

2 But see May (2014) for a critique of the philosophical significance of the purported relation between disgust and moral judgment.

3 We will subsequently refer to ‘COVID-19’ simply as ‘Covid’.

4 Our account of tuberculosis in this section draws heavily on Tomes (1997).

5 For a short history, see: https://nf.lung.ca/about-us/our-history/cross-lorraine.

6 The much-discussed Speaker incident in 2007 provides an interesting case study; moralization may have been warranted, to the extent that Andrew Speaker travelled and risked infecting others with extensively drug-resistant tuberculosis (XDR-TB), except it turns out that the facts were wrong—Andrew speaker did not have XDR-TB in the end (Selgelid 2008).

7 We discuss conditions for moral responsibility and moral blame more formally in the following section.

8 We address the issue of spreading infection in the next section about COVID-19 vaccination status.

9 From the perspective of government, there are also important moral reasons why there should be space for people to make other-regarding choices (cf. Kraaijeveld 2021b).

10 The point that we want to emphasize is not so much any specific number, but rather that moralization of the issue can obscure the facts and give rise to beliefs based on unrealistic estimates. A good, honest public discussion about vaccine passports must address the question of whether the moral costs of excluding so many citizens from society is worth the NNE—whatever that number may be, based on the best available data.

11 Thanks to an anonymous reviewer for raising this point.

12 It should be noted that scientists are not immune from moralization, so that potential mismoralization within the scientific community may pose its own set of problems (e.g., when it comes to peer review, considerations for the (social) acceptability of publication, and so on).

13 A potentially fruitful way of studying (mis)moralization and technology would be through an experimental philosophical approach (see Kraaijeveld 2021c).
